# Successful pregnancy after operation in an infertile woman caused by luteinizing hormone-secreting pituitary adenoma: case report and literature review

**DOI:** 10.1186/s12902-020-00677-3

**Published:** 2021-01-12

**Authors:** Yi Zhang, Cheng Chen, Min Lin, Kan Deng, Huijuan Zhu, Wenbin Ma, Hui Pan, Renzhi Wang, Yong Yao

**Affiliations:** 1 Department of Neurosurgery, Peking Union Medical College Hospital, Chinese Academy of Medical Sciences and Peking Union Medical College, No.1 Shuaifuyuan Wangfujing Dongcheng District, Beijing, 100730 China; 2 Department of Endocrinology, Peking Union Medical College Hospital, Chinese Academy of Medical Sciences and Peking Union Medical College, Beijing, China

**Keywords:** Luteinizing hormone-secreting pituitary adenoma, Infertility, Operation, Case report

## Abstract

**Background:**

Functional gonadotroph adenomas (FGAs) are rare adenomas that most commonly secrete FSH. However, solitary LH-secreting pituitary adenomas are unusual.

**Case presentation:**

A 30-year-old woman with elevated LH and normal FSH presented with inability to conceive. An MRI revealed an enlarged sella turcica and an intrasellar mass. Treatment with transsphenoidal resection led to normalization of LH and estradiol, as well as successful pregnancy. And we reviewed 6 cases of LH-secreting pituitary adenomas from 1981 to 2020.

**Conclusions:**

Our case is unique because of the LH-secreting pituitary adenoma without FSH hypersecretion. This case indicates that pituitary adenoma should be considered when other diseases causing infertility have been excluded.

## Background

Functional gonadotroph adenomas (FGAs) are adenomas secreting biologically active gonadotropins and causing distinct clinical manifestations. Clinically FGAs are rare and their exact prevalence is not known. They most commonly secrete FSH, less frequently secrete both FSH and LH together, and LH alone is unusual. And we review 6 cases of LH-secreting pituitary adenomas. Several reports indicate that LH-secreting pituitary adenoma may cause neurologic symptoms and acquired hypogonadism or precocious puberty. In our case, we report infertility as the first manifestation, and she successfully conceived after the operation.

## Case presentation

**Fig. 1:**
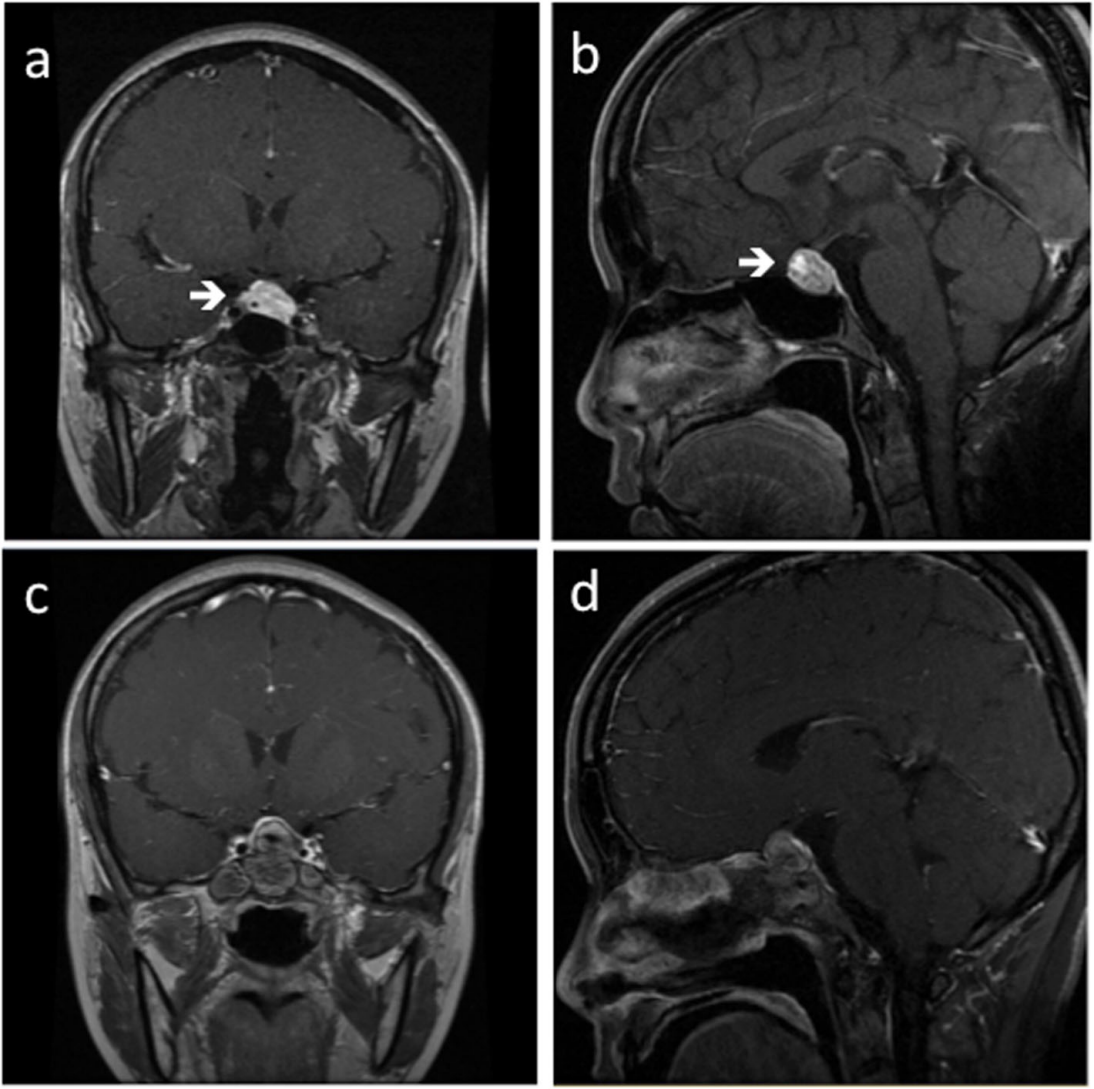
**a** Coronal view of cranial contrast-enhanced MRI before operation (white arrow). **b** Sagittal view of cranial contrast-enhanced MRI before operation (white arrow). **c** Coronal view of cranial contrast-enhanced MRI of this patient in 3 months after operation. **d** Sagittal view of cranial contrast-enhanced MRI of this patient in 3 months after operation

A 30-year-old woman presented to the local hospital complaining of erratic periods and inability to conceive for 2 years. She had serum gonadotropins measured; LH was raised, but not FSH, estradiol or prolactin (Chemiluminescent Immunoassay, Beckman DxI800). Both transvaginal and pelvic ultrasonography were normal. 7 months later, the LH level of the patient was still high at 37.20 IU/L (follicle stage, normal 2.12–10.89 IU/L), and FSH level of 7.30 IU/L (follicle stage, normal < 10 IU/L), PRL level of 21.32 μg/L (normal < 30 μg/L), progesterone level of 0.88 ng/mL (follicle stage, 0.38–2.28 ng/mL), testosterone level of 0.35 ng/L (normal 0.10–0.75μg/L), estradiol level of 72.35 pg/ml (follicle stage, normal < 27–122 pg/ml). The patient was placed on a trial of contraceptive pills (drospirenone and ethinylestradiol tablets) for 3 months. Her LH remained elevated at 42.24 IU/L. An MRI study of the pituitary revealed an enlarged sella turcica and an intrasellar mass (Fig. 1a, b).

She was referred to our hospital for further diagnosis and treatment. Repeat laboratory tests showed elevated LH level of 45.6 IU/L and elevated estradiol level of 266.3 pg/ml, with normal FSH, P, PRL, GH, IGF1, T3, T4, F, and ACTH levels. She refused to accept a GNRHa test. The patient experienced menarche at 14 years of age. Her menstrual intervals were erratic over the past several years. She is a teacher in a local middle school. She denied other significant medical history, such as smoking, use of alcohol and substance abuse. On physical examination, the patient weighed 51 kg and was 156 cm tall (body mass index [BMI] 21.0 kg/m^2^). The ophthalmological test showed normal. She appeared as normally developed female.

**Fig. 2:**
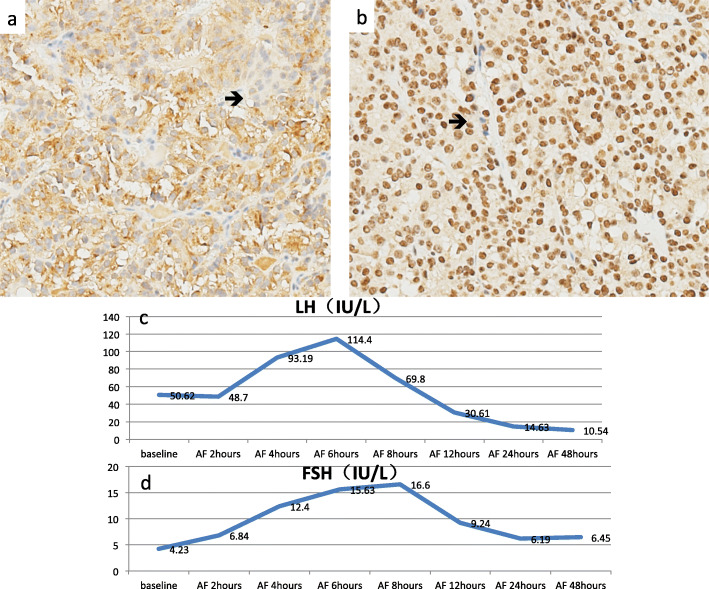
**a** Immunohistochemical staining of the tumor shows a positive result of LH (original magnification × 100) (black arrow). **b** Immunohistochemical staining of the tumor shows a negative result of FSH (original magnification × 100) (black arrow). Usually a positive signal of FSH shows in the cytoplasm, but in our biopsy the signal shows in the nuclear, so it should also be considered as a negative result. **c** The concentration of serum LH peaks at 6 h after the operation and gradually falls to normal level. **d** The concentration of serum FSH peaks at 8 h after the operation and gradually falls to normal level

The patient underwent transsphenoidal resection of pituitary mass without complications. The tumor measured 2.2*1.3*1.5 cm, and the immunohistochemical staining of the tumor was positive for LH, partially positive for GH, and negative for FSH, TSH, ACTH, P53 (Fig. 2a, b). After operation, laboratory tests revealed LH level normalized in 24 h, and estradiol level normalized in 48 h. However, our patient demonstrated an unanticipated increase in LH and FSH after the operation, with peaks at 6 h for the former and 8 h for the latter (Fig. 2c, d). Due to normalization of LH and estradiol, she resumed menstruation in 16 days and successfully became pregnant 3 months after the operation, at which point, she was still under follow-up in our clinic. The patient was very satisfied with the operation and the prognosis by MRI (Fig. 1c, d).

## Discussion and conclusions

Gonadotroph adenomas are relatively common among pituitary adenomas, accounting for 25–35% of all macroadenomas recognized clinically, and 43–64% of silent adenomas [1]. They are defined by immunohistochemically positive for FSH, LH and/or their subunits even when there is no measurable hormone section. Amongst gonadotroph adenomas, most are non-functional and only a minority are functional gonadotroph adenomas (FGAs) that secrete biologically active gonadotropins. Even for those hormone-producing adenomas the hormonal production is usually not high enough to cause recognizable symptoms besides common neurological symptoms resulted from mass effect. This leads to difficulty in recognizing gonadotroph adenomas.

FGA is a rare disease. Only small case series or individual case reports have been documented. The tumors usually produce FSH, less frequently both FSH and LH together. In contrast, from 1981 to 2020, only 6 cases of LH-secreting adenomas have been reported in the English literature, with or without free alpha-subunits [2–7] (Table 1), illustrating the rarity of this disease and the difficulty in diagnosis based on clinical symptoms. FGA are difficult to be recognized because of the poorly so-called “silent”. Although these adenomas do produce the complete or partial gonadotropins, they don’t raise the serum gonadotropin concentrations [7]. So, it is important and essential to detect biochemical testing in pituitary adenomas by their excessive hormonal secretion.

**Table 1:** Cases of LH-secreting adenomas reported in the English literature

Study	Age	gender	Symptoms	Tumor	LH (mIU/mL)	a-subunit (ng/ml)	FSH (mIU/mL)	E2(pg/ml)	T (ng/ml)	Medical therapy	Operation
Peterso [2]	30	Male	azzospermic	Intrasellar mass	207 (6–26)	72(< 0.5–2.5)	2(normal range 5–25)	110(< 10–60)	1500 (300–1000)	TRH, LRH, L-dopa, metyrapone,	Transsphenoidal operation
Klibanski [3]	48	Male	Increase in libido, blind	Pituitary tumor	99 (2–18)	5.7(< 2.5)	5.5(−)	219(< 52)	1660 (300–1100)		Transfrontal craniotomy
Vos [4]	20	Male	Grand-mal seizures	Hypothalamic-pituitary tumor	140 (2–15)	9105 (3–30)	4 (3–12)		53 (12–30)	SMS201–995	Transsphenoidal operation
Castelo-Branco [5]	31	Female	OHSS	Pituitary macroadenoma	4.9(−)		7.2(−)			Prolactin, cabergoline, somatostatin	Transsphenoidal operation
Roman [6]	33	Male	Testicular pain, dysuria	Intrasellar mass	207 (3–9)	72(< 0.5–2.5)	2 (4–11)		15,000 (30000–80,000)	LHRH	Transsphenoidal operation
Kadakia [7]	9	Male	Precocious puberty	Anterior pituitary tumor	5(< 0.49)		< 0.05 (0.87–9.16)		44,200(< 4200)	leuprolide	Transsphenoidal operation

*LH* Luteinizing Hormone, *FSH* Follicle stimulating hormone, *E2* estradiol, *T* Testosterone, *SMS* somatostatin, *OHSS* ovarian hyperstimulation syndrome

Gonadotroph adenomas are equally common between genders according to in vitro evidence, but clinically most of them appear in men. This may partially due to the simpler diagnosis of male patient. Our study reveals that 5 of the 6 individual cases were men ranging from 9 to 48 years old, on the basis of neurologic symptoms and acquired hypogonadism or precocious puberty. The only female patient among the 6 cases presented with ovarian hyperstimulation syndrome (OHSS). The thickened endometrium and/or recruitment of multiple dominant follicles on pelvic ultrasound are usually caused by FSH adenomas [5]. However, the female patient in this case has relatively normal FSH, which may explain the normal pelvic ultrasound findings. In some postmenopausal women, a gonadotroph adenoma that secretes intact gonadotropins would not result in obvious pelvic changes, because gonadotropin levels are already high and a postmenopausal ovary cannot be stimulated to produce follicles or estradiol.

This female patient merely presents with infertility, while most common presenting clinical manifestation of premenopausal women include menstrual irregularity, spontaneous vaginal spotting, galactorrhea and mass effect. Female infertility can result from ovulation disorders, uterine abnormalities, tubal obstruction, and peritoneal factors. In contrast, infertility due to gonadotroph pituitary adenomas is rare. There are less than 5 literatures documenting FSH-secreting adenomas causing infertility [8]. Herein, we report the first case of infertility caused by LH-secreting pituitary adenoma, without FSH-secreting. The rareness of FGA explains why infertile female will not be considered as pituitary adenomas on first impressions. According to our case, gonadotroph adenoma should be considered in a reproductive-aged woman who presents with infertility, and elevated LH and/or FSH, after elusive of other common disease that can cause infertility. And a pituitary imaging is necessary to make the diagnosis.

Diagnosis of LH-secreting pituitary adenoma was demonstrated by 1) elevated serum LH and estradiol concentrations; 2) normalization of serum LH and successfully pregnant after pituitary adenomectomy; 3) immunohistochemical staining positive for LH. However, GH was normal in serum, whereas immunohistochemical staining of GH was partially positive. This may own to combining with NFPAs. A study of 103 patients with NFPA showed 18 patients were immunohistochemically positive for both FSH and LH, one patient was positive for both GH and LH. According to the new WHO classification, several transcription factors and other differentiation driving factors played key roles in adenohypophysis, such as PIT-1, SF-1, T-PIT and so on. With this new paradigm, the 2017 WHO classification classifies adenomas according to their pituitary cell lineage rather than the hormone-secreting pituitary adenoma.

There are few systemic series on the optimal management of FGA, and the relevant data relies on case reports or very small cases. Surgical removal of the adenoma remains the optimal approach and, if successful, leads to restoration of normal gonadotropin secretion, regular menstruation, and resolution of infertility. However, after the operation, there was a peak of serum LH and FSH curve in 24 h, which may own to the excess LH and FSH secreting into blood of the adenoma pituitary irritated by operation. The most typical endocrinological abnormality in our case was the elevation of LH and estradiol, and FSH within normal limit. Our study revealed 3 of 6 LH-secreting adenomas were with decreased FSH, may due to the impairment by the adenoma of FSH secreting from the normal pituitary, and 3 of them were with normal FSH. However, in FSH-secreting adenomas, elevated FSH was always accompanied by decreased LH [9].

One limit we have to acknowledge is that serum and immunostaining of alpha-subunit were not measured owning to the limit of detection technology, and we cannot make a conclusion whether the pure LH-secreting adenoma was accompanied by free alpha-subunit hypersecreted.

In our case, a woman with infertility as the first manifestation successfully conceived after pituitary mass resection. This case indicates that pituitary adenoma should be considered when other diseases causing infertility have been excluded.

## Data Availability

All the data generated and/or analyzed during this study are included in this published article.

## Abbreviations

FGAs: Functional gonadotroph adenomas
FSH: Follicle-stimulating hormone
LH: Luteinizing hormone
MRI: Magnetic resonance imaging
PRL: Prolactin
T: Testosterone
P: Progesterone
F: Cortisol
GH: Growth hormone
IGF1: Insulin-like growth factor 1
ACTH: Adrenocorticotropic Hormone
GNRHa: Gonadotropin-releasing hormone agonist
BMI: Body mass index
OHSS: Ovarian hyperstimulation syndrome
NFPAs: Non-functional pituitary adenomas
E2: Estradiol
